# Four Novel p.N385K, p.V36A, c.1033–1034insT and c.1417–1418delCT Mutations in the Sphingomyelin Phosphodiesterase 1 (*SMPD1*) Gene in Patients with Types A and B Niemann-Pick Disease (NPD)

**DOI:** 10.3390/ijms16046668

**Published:** 2015-03-24

**Authors:** Masoumeh Dehghan Manshadi, Behnam Kamalidehghan, Fatemeh Keshavarzi, Omid Aryani, Sepideh Dadgar, Ahoora Arastehkani, Mahdi Tondar, Fatemeh Ahmadipour, Goh Yong Meng, Massoud Houshmand

**Affiliations:** 1 Department of Biology, Science and Research Branch, Islamic Azad University, Kurdistan 6614996164, Iran; E-Mails: m_dehghanmanshadi@yahoo.com (M.D.M.); ahoora_mazda_930@yahoo.com (A.A.); 2 Department of Pharmacy, Faculty of Medicine, University of Malaya (UM), Kuala Lumpur 50603, Malaysia; E-Mails: kamalidehghan.behnam@gmail.com (B.K.); ahmadipourf@gmail.com (F.A.); 3 Department of Biology, Sanandaj Branch, Islamic Azad University, Kurdistan 6616935391, Iran; E-Mail: gol.keshavarzi@gmail.com; 4 Medical Genetics Department, Special Medical Center, Tehran 1599666615, Iran; E-Mails: o_aryani@yahoo.com (O.A.); sepideh.dadgar@gmail.com (S.D.); 5 Department of Biochemistry and Molecular & Cellular Biology, Georgetown University, Washington, DC 20057, USA; E-Mail: mt969@georgetown.edu; 6 Department of Animal Science, Faculty of Veterinary Medicine, Universiti Putra Malaysia, Serdang 43400, Malaysia; E-Mail: gohyongmeng@gmail.com; 7 Medical Genetics Department, National Institute for Genetic Engineering and Biotechnology (NIGEB), Tehran 1497716316, Iran

**Keywords:** types A and B niemann-pick disease (NPD), sphingomyelin phosphodiesterase 1 (*SMPD1*) gene, acid sphingomyelinase (ASM), p.G508R, p.N385K and p.V36A, c.1033–1034insT and c.1417–1418delCT

## Abstract

Background: Types A and B Niemann-Pick disease (NPD) are autosomal-recessive lysosomal storage disorders caused by the deficient activity of acid sphingomyelinase due to mutations in the sphingomyelin phosphodiesterase 1 (*SMPD1*) gene. Methods: In order to determine the prevalence and distribution of *SMPD1* gene mutations, the genomic DNA of 15 unrelated Iranian patients with types A and B NPD was examined using PCR, DNA sequencing and bioinformatics analysis. Results: Of 8 patients with the p.G508R mutation, 5 patients were homozygous, while the other 3 were heterozygous. One patient was heterozygous for both the p.N385K and p.G508R mutations. Another patient was heterozygous for both the p.A487V and p.G508R mutations. Two patients (one homozygous and one heterozygous) showed the p.V36A mutation. One patient was homozygous for the c.1033–1034insT mutation. One patient was homozygous for the c.573delT mutation, and 1 patient was homozygous for the c.1417–1418delCT mutation. Additionally, bioinformatics analysis indicated that two new p.V36A and p.N385K mutations decreased the acid sphingomyelinase (ASM) protein stability, which might be evidence to suggest the pathogenicity of these mutations. Conclusion: with detection of these new mutations, the genotypic spectrum of types A and B NPD is extended, facilitating the definition of disease-related mutations. However, more research is essential to confirm the pathogenic effect of these mutations.

## 1. Introduction

Type A Niemann-Pick disease (NPA, MIM# 257200) is a severe neurodegenerative disease of infancy associated with advanced psychomotor, hepatosplenomegaly, and death that typically occurs in the first three years of life [1,2,3]. However, type B Niemann-Pick disease (NPB, MIM# 607616) is a late-onset non-neuronopathic disease that is correlated with hepatosplenomegaly and respiratory problems, where the majority of patients survive until adulthood [4]. Types A and B Niemann-Pick disease are autosomal-recessive sphingolipidosis caused by mutations in the sphingomyelin phosphodiesterase 1 gene (*SMPD1*, MIM# 607608, GenBank# M81780.1) resulting in lysosomal acid sphingomyelinase (ASM, E.C. 3.1.4.12) malfunction. The *SMPD1* gene is located on chromosome 11 at p15.1–p15.4, is 5-Kbp in length and is composed of six exons encoding 587 amino acids. Niemann-Pick disease (NPD) is characterized by the intracellular accumulation of sphingomyelin in the liver, spleen, lungs, bone marrow, or brain, resulting in the presence of foamy macrophages in these organs [5,6]. ASM deficiency is rare with an estimated incidence of 0.4–0.6 in 100,000 newborns [7]. The human ASM protein is synthesized as a 75-kDa, glycosylated prepolypeptide, which is converted to a 72-kDa precursor form. The precursor is subjected to two different processing events. A minor portion is cleaved in the endoplasmic reticulum-Golgi complex, yielding a 57-kDa form, and the majority is processed into a 70-kDa mature form [8]. To date, over 100 mutations that cause acid sphingomyelinase deficiency have been reported [9].

Recognizing the common mutations in *SMPD1* that lead to NPD in Iran can help prenatal diagnosis in families at risk for infants with NPD and enzyme therapy in children with NPD prior to the onset of severe symptoms [10]. In this study, we examined *SMPD1* mutations in Iranian patients affected by types A and B NPD.

## 2. Results

**Figure 1:**
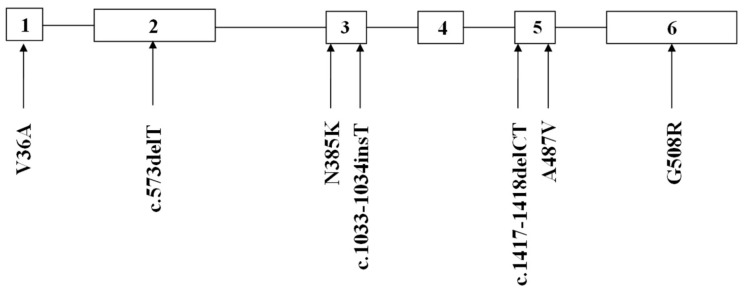
The mutations in the *SMPD1* gene of Iranian patients with types A and B Niemann-Pick disease (NPD) as determined in this study.

**Figure 2:**
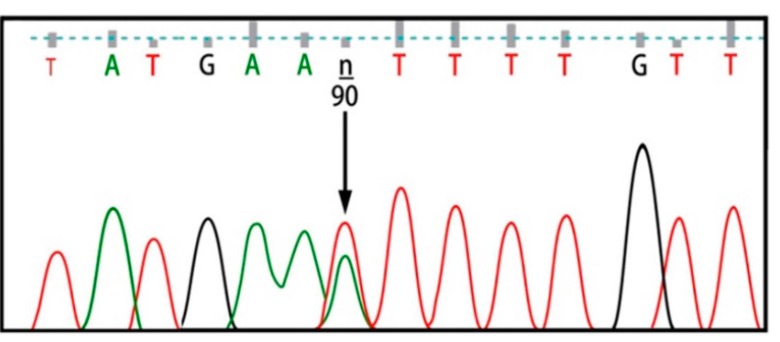
p.N385K heterozygous mutation.

**Figure 3:**
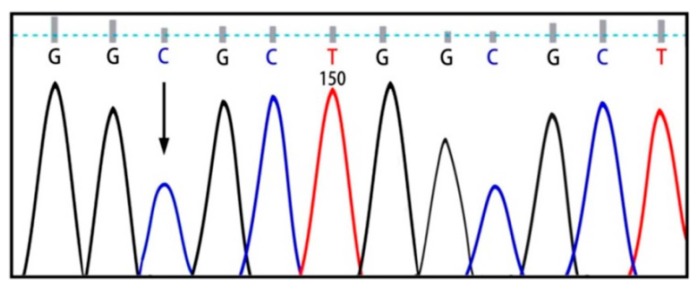
p.V36A homozygous mutation.

**Figure 4:**
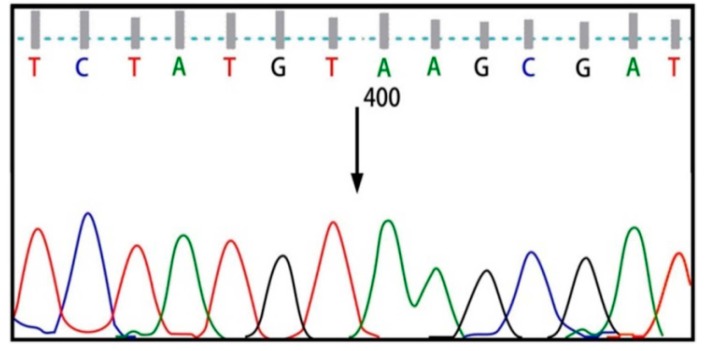
T insertion mutation at codon 345.

**Figure 5:**
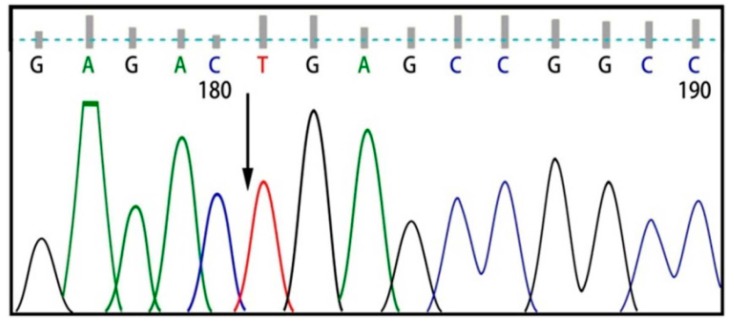
CT deletion mutation at codon 473.

Six exons of the *SMPD1* gene were analyzed from 15 patients affected by types A and B NPD, as summarized in Table 1 and Figure 1. Here, 8 patients showed the p.G508R mutation in exon 6, including 5 patients (P1, P11, P22, P26 and P35) with homozygous mutations and 3 patients (P3, P24 and P28) with heterozygous mutations (Table 1). One patient (P8) showed heterozygous p.N385K and p.G508R mutations (Figure 2). One patient (P31) showed heterozygous p.A487V and p.G508R mutations. Two patients showed p.V36A missense mutation, in which one (P5) proved to be homozygous and the other (P12) heterozygous (Figure 3). One patient (P15) showed a homozygous c.1033–1034insT mutation (Figure 4). One patient (P32) showed a homozygous c.573delT mutation. One patient (P29) showed a homozygous c.1417–1418delCT mutation (Figure 5). The results of bioinformatics analysis indicated that two new p.V36A and p.N385K mutations, as well as the p.G508R mutation, decreased the ASM protein stability (Table 2).

**Table 1:** Type of mutations and ASM activity found in 15 Iranian patients with types A and B NPD disease.

No.	Patients No.	Leukocytes-ASM Activity (nmol·17 h^−1^·mg^−1^)	Exon	Mutation
1	P1	4.83	6	p.G508R homozygous
2	P11	4.56	6	p.G508R homozygous
3	P22	3.73	6	p.G508R homozygous
4	P26	5.45	6	p.G508R homozygous
5	P35	5.15	6	p.G508R homozygous
6	P3	4.33	6	p.G508R heterozygous
7	P24	3.78	6	p.G508R heterozygous
8	P28	5.08	6	p.G508R heterozygous
9	P31	4.29	5, 6	p.A487V, p.G508R compound heterozygosity
10	P8	4.11	3, 6	p.N385K *, p.G508R compound heterozygosity
11	P5	3.66	1	p.V36A * homozygous
12	P12	3.93	1	p.V36A * heterozygous
13	P15	4.25	3	c.1033–1034insT * homozygous
14	P32	4.29	2	c.573delT homozygous
15	P29	5.88	5	c.1417–1418delCT * homozygous

* shows new mutation.

**Table 2:** Bioinformatics analysis of three p.G508R, p.N385K and p.V36A mutations using I-mutant 2.0 software (Bologna Biocomputing Group, Bologna, Italy). Prediction of protein stability changes based on 3 mutations—p.G508R, p.N385K and p.V36A—indicates decrease in protein stability.

Mutation	Bioinformatic Analysis (I-Mutant 2.0)
	Prediction of Protein Stability
G508R	decrease stability
N385K	decrease stability
V36A	decrease stability

## 3. Discussion

Patients with enzyme acid sphingomyelinase (ASM) deficiency are classified as the hallmark of having types A or B Niemann-Pick disease. The degree of clinical involvement largely depends on the type of SMPD1 mutations inherited and the effect of these mutations on the residual ASM polypeptide. In this study, the *SMPD1* mutations were investigated in Iranian patients affected by types A and B NPD.

Our findings are consistent with previous studies showing a deficiency in ASM enzyme activity in NPD patients types A [11], and B [12]. However, decrease in lysosomal stability can be effectively corrected by treatment with recombinant Hsp70 [13]. Another investigation indicated mutations in the *SMPD1* gene [14] that seemed to decrease the residual ASM activity by producing a modified ASM polypeptide [15,16]. Diagnosis of the types of NPD and also the severity of disease in NPB patients based on residual ASM activity is not always possible due to defects in ASM activity measuring methods and the existence of ASM activity in hematopoietic cell homogenates [17]. Therefore, diagnosing the types and severity of the disease in NPD patients according to genetic testing is of outmost importance. In this study, five patients (P1, P11, P22, P26 and P35) and 3 patients (P3, P24 and P28) showed respectively homozygous and heterozygous p.G508R mutation in exon 6, while all parents of these patients for the p.G508R mutation were heterozygous. The p.G508R mutation was reported for the first time by Mussig [18], in a patient with the clinical profile of NPD, whereas the pathogenicity of p.G508R mutation has not been elucidated.

A patient (P31) was heterozygous for the p.G508R mutation and heterozygous for the p.A487V mutation, which is a condition of having two heterogeneous recessive alleles at a particular locus (compound heterozygosity), which can play the role of a homozygous mutation and lead to a genetic disease. The p.A487V mutation that was observed in our patient (P31) was reported by Simonaro and colleagues (2006), where the pathogenicity of this mutation has already been proven [19].

Both heterozygous p.G508R and p.N385K mutations were detected in exons 6 and 3 of patient (P8), respectively. The p.N385K mutation was reviewed using the I-Mutant 2.0 and the MUpro softwares, and result analysis demonstrated the effect of this mutation in reducing ASM stability. Although the p.G508R and p.N385K mutations are located on different exons, they may have the potentiality to act as a compound heterozygote to cause NPD. Additionally, bioinformatics analysis of a new homozygous p.V36A mutation in a patient (P5) using the I-Mutant 2.0 and the MUpro softwares demonstrated that p.V36A reduces ASM stability. 

Other than the single nucleotide changes (missense mutations) in this study, a number of insertion and deletion mutations were also detected in patients, including P15 and P32, respectively. A patient (P15) revealed a new homozygous c.1033–1034insT mutation of the *SMPD1* gene which leads to the disassembling of the reading frame of the ASM polypeptide, leading to induction of a fundamental modification in the structure and function of the ASM enzyme. Parents of patient P15 were both heterozygous for this mutation, which confirms the recessive inheritance pattern of the disease. Patient P32 showed a homozygous c.573delT mutation, spotted in the form of CCAAA^^19^CCCtAGC. This delT mutation was identified by Gluck and colleagues (1997) before [20]. Moreover, patient P29 showed a new homozygous c.1417–1418delCT mutation. However, the delCT mutation could not be identified in parents of the patient due to inaccessibility.

## 4. Experimental Section

### 4.1. Ethics Statement

Blood samples from 15 unrelated patients with types A and B Niemann-Pick disease and 40 healthy individuals as controls were obtained from the Medical Genetics Department of the Special Medical Centre (SMC), Tehran-Iran, between January 2007 and November 2011. In this study, the peripheral leukocytes-ASM activity (Table 1) in all NPD patients were below the normal range (9.5–58 nmol·17 h^−^^1^·mg^−^^1^). Documented, written consent was obtained from the patients, as approved for the entire study protocol by the SMC governing ethics committee at the time. 

### 4.2. DNA Extraction and Primer Design

QIAamp kit (QIAamp*^®^* DNA Micro Kit *#*56304, QIAGEN, Hilden, Germany) was used for the isolation of DNA from the blood samples according to the manufacturer’s protocol. Primers were designed (Table 3) using the software GENE RUNNER Version 3.4.0.0 (Hasting Software Inc., Hastings, NY, USA). 

**Table 3:** Five pairs of primers were applied for the detection of point mutations in the exon and exon-intron junction regions of the *SMPD1* gene.

Exons	Primer Sequence (5' to 3')	Product Size (bp)	Temperature(°C)
E1	F: GAGGGCTGGCTAGGGTCCAG	440	68
	R: CCAGCCCCAGCAGTCCTT		
E2	F: TCCTCTGCTCTGCCTCTGATTTCTCACCAT	900	68
	R: AATCAGAGACAATGCCCCAGGTTCCCTTCT		
E3	F: GGAGGACCAGGATTGGAACA	300	62
	R: CAGAGGGGCGCCAGCTCAAC		
E4	F: GATTCAGCTCATGGTCACTG	300	62
	R: GGATGGTGAGATGCTCAAGG		
E5, 6	F: GCATCTCACCATCCCTGTTGTCCCATG	1000	63
	R: CTGTTTCACCCTTTCCTACATCAAGAACT		

### 4.3. Polymerase Chain Reaction (PCR)

PCR was carried out using five sets of primers (Table 3). Briefly, PCR was carried out in a final volume of 25 µL containing 100–200 ng of total DNA, 10 pmol of each primer, 2.5 mM MgCl_2_, 200 mM each of dNTP and 1 U of Taq DNA polymerase (Roche Diagnostics, Mannheim, Germany). The reaction mixture was cycled 35 times at 95 °C for 1 min, annealing temperature (°C) for 1 min (refer to Table 3) and 72 °C for 1 min. PCR products were separated on 2% agarose gels, run in 0.5× TBE at 110 V for 50 min, stained in 0.002 mg/mL ethidium bromide and visualized using means of UV light.

### 4.4. DNA Sequencing

The PCR products were sequenced with the forward or reversed primers on a ABI 3700 sequencer (Kosar Company, Tehran, Iran) and compared with control samples using the FinchTV program and analyzed on the NCBI website (available online: http://blast.ncbi.nlm.nih.gov/Blast.cgi). With these methods, the target sequence for each patient were compared with the normal reference sequence, and mutations in the exons and the splicing sites of the introns in the *SMPD1* gene in our samples were determined.

### 4.5. Bioinformatics Analysis

Bioinformatics analysis were performed using the I-Mutant 2.0 (available online:  http://folding.uib.es/i-mutant/i-mutant2.0.html) and the MUpro (available online: http://mupro.proteomics.ics.uci.edu/) in order to check the effect of new mutations on ASM stability in the *SMPD1* gene.

## 5. Conclusions

In conclusion, with finding of these novel mutations, the genotypic spectrum of types A and B Niemann-Pick disease (NPD) patients has been extended and could probably facilitate the definition of pathogenic disease-related mutations in human disease.

## Abbreviations

NPD: Niemann-Pick disease
ASM: acid sphingomyelinase
SMC: Special Medical Center
SMPD1: sphingomyelin phosphodiesterase 1
